# Multilocus genetic profile in dopaminergic pathway modulates the striatum and working memory

**DOI:** 10.1038/s41598-018-23191-y

**Published:** 2018-03-29

**Authors:** Chao Wang, Bing Liu, Xiaolong Zhang, Yue Cui, Chunshui Yu, Tianzi Jiang

**Affiliations:** 1 Shenzhen Key Laboratory of Affective and Social Cognitive Science, College of Psychology and Sociology, Shenzhen University, Shenzhen, 518060 China; 2 Key Laboratory for NeuroInformation of Ministry of Education, School of Life Science and Technology, University of Electronic Science and Technology of China, Chengdu, 610054 P. R. China; 3 Brainnetome Center, Institute of Automation, Chinese Academy of Sciences, Beijing, China; 4 National Laboratory of Pattern Recognition, Institute of Automation, Chinese Academy of Sciences, Beijing, China; 5 CAS Center for Excellence in Brain Science, Institute of Automation, Chinese Academy of Sciences, Beijing, China; 6 Department of Radiology, Tianjin Medical University General Hospital, Tianjin, China; 7 The Queensland Brain Institute, University of Queensland, Brisbane, Australia

## Abstract

Dopamine is critical in pathophysiology and therapy of schizophrenia. Many studies have reported altered dopaminergic activity in the dorsal but not ventral striatum in schizophrenia. Based on the largest genome-wide association study of schizophrenia to date, we calculated the polygenic risk score (PGRS) of each subject in a healthy general group, including all variations in the set of functionally related genes involved in dopamine neurotransmitter system. We aimed to test whether the genetic variations in the dopaminergic pathway that have been identified as associated with schizophrenia are related to the function of the striatum and to working memory. We found that a higher PGRS was significantly associated with impairment in working memory. Moreover, resting-state functional connectivity analysis revealed that as the polygenic risk score increased, the connections between left putamen and caudate and the default mode network grew stronger, while the connections with the fronto-parietal network grew weaker. Our findings may shed light on the biological mechanism underlying the “dopamine hypothesis” of schizophrenia and provide some implications regarding the polygenic effects on the dopaminergic activity in the risk for schizophrenia.

## Introduction

Understanding the dopamine neurotransmitter system is critical for research on the therapy and pathophysiology of schizophrenia^1,2^. Enhanced striatal dopamine neurotransmission^3^ and prefrontal cortex dysfunction^4^ are consist findings in schizophrenia that might correlated to functional impairments and cognitive deficits in schizophrenia^5^, and the striatal dopamine D2 receptor is the therapeutic target of most antipsychotic drugs^5^. Many pathophysiological models of mental illness ascribe a key role to the striatum (the caudate nucleus and putamen) because of its rich connectivity with other regions known to be dysfunctional in psychiatry, especially the prefrontal cortex^6^. Early animal studies have emphasized dysfunction of the mesolimbic dopaminergic pathway, connecting ventral striatum (the nucleus accumbens) with medial prefrontal cortex^7^. However, subsequent *in vivo* PET research has indicated that patients with schizophrenia show up-regulation of dopaminergic activity in the dorsal but not ventral striatum^3^.

Working memory deficits area central feature of cognitive impairment in schizophrenia^8^, and some functional magnetic resonance imaging studies have found that patients with schizophrenia showed not only alterations in prefrontal activity during working memory processing^9,10^ but also abnormalities in striatal function and dysfunction of dopamine signaling, which are both possible explanations for working memory deficits^5,11^.

In addition, schizophrenia is highly heritable, but the specific genetic variants identified have very minor effects. This implies that a large number of genetic variants may have additive effects on the complex phenotypes, i.e., that schizophrenia is also a polygenic inheritance disorder^12,13^. By using genome-wide association studies (GWAS), we can calculate a polygenic risk score (PGRS), which involves a few significant SNPs from GWAS and SNPs that meet nominal significance criteria but fail to reach the genome-wide significance level, for each individual associated with a particular disorder to capture its polygenic nature^13,14^. The PGRS can also be used to estimate the cumulative genetic risk for the disease in diverse phenotypes^15,16^. Resting-state functional connectivity is an effective method for detecting the coherent patterns of spontaneous neural activity fluctuations. It is under strong genetic control and reflects the basis of brain functional organization^17,18^. Thus, imaging genetics could help us to investigate the additive effects of genetic risk variants on brain function.

In this study, the PGRS of schizophrenia was derived from the largest GWAS study in schizophrenia^19^, and we only included all variations in the set of functionally related genes involved in dopamine functioning. We aimed to dive deeper into the relationships between the PGRS related to dopamine genes and the structure and function of the dorsal striatum as well as working memory performance. We hypothesized that the dopamine gene-related polygenic risk would predict altered function in the dorsal dopaminergic pathway.

## Results

### The relationships of dopamine gene-related PGRS with working memory performance

The dopamine gene-related PGRS showed a significant negative correlation with the 3-back working memory correction rate (r = −0.122, p = 0.043), whereas the anti-correlation with 2-back working memory performance (r = −0.045, p = 0.462) did not reach statistical significance in the partial correlation analysis. Thus, the PGRS is a reliable indicator of individual working memory performance.

### The relationships of dopamine gene-related PGRS with functional connectivity of the striatum

**Figure 1:**
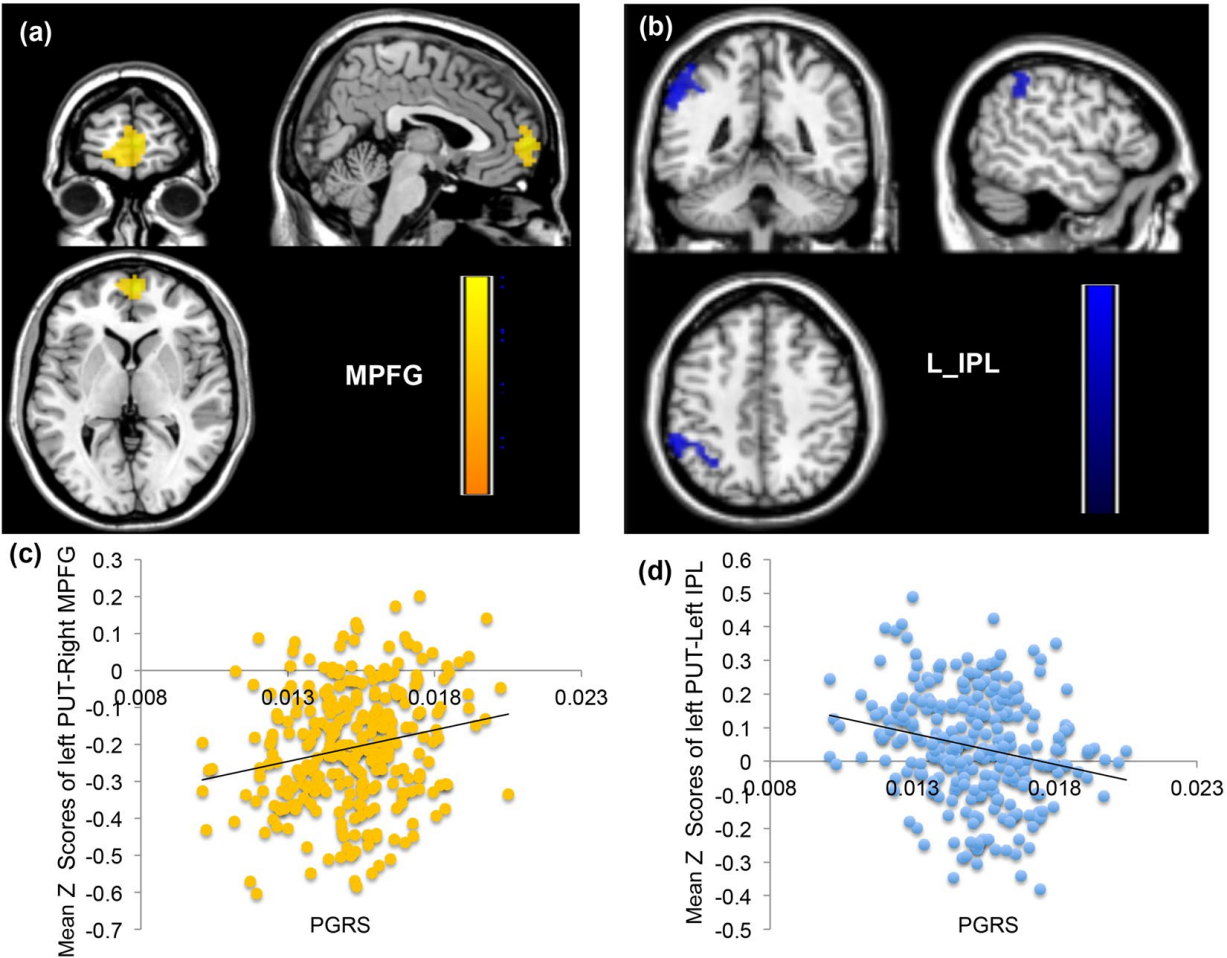
Association of the dopamine gene-related PGRS and functional connectivity with the putamen. (**a**) and (**c**) show that the functional connectivity between putamen (PUT) and medial prefrontal cortex (MPFG) increases with higher PGRS values (r = 0.214, p = 3.62 × 10^−4^); (**b**) and (**d**) show that connections between putamen (PUT) and inferior parietal lobule (IPL) are attenuated as the PGRS values increase (r = −0.219, p = 2.59 × 10^−4^) (Alphasim corrected p < 0.05, clusters > 60).

**Figure 2:**
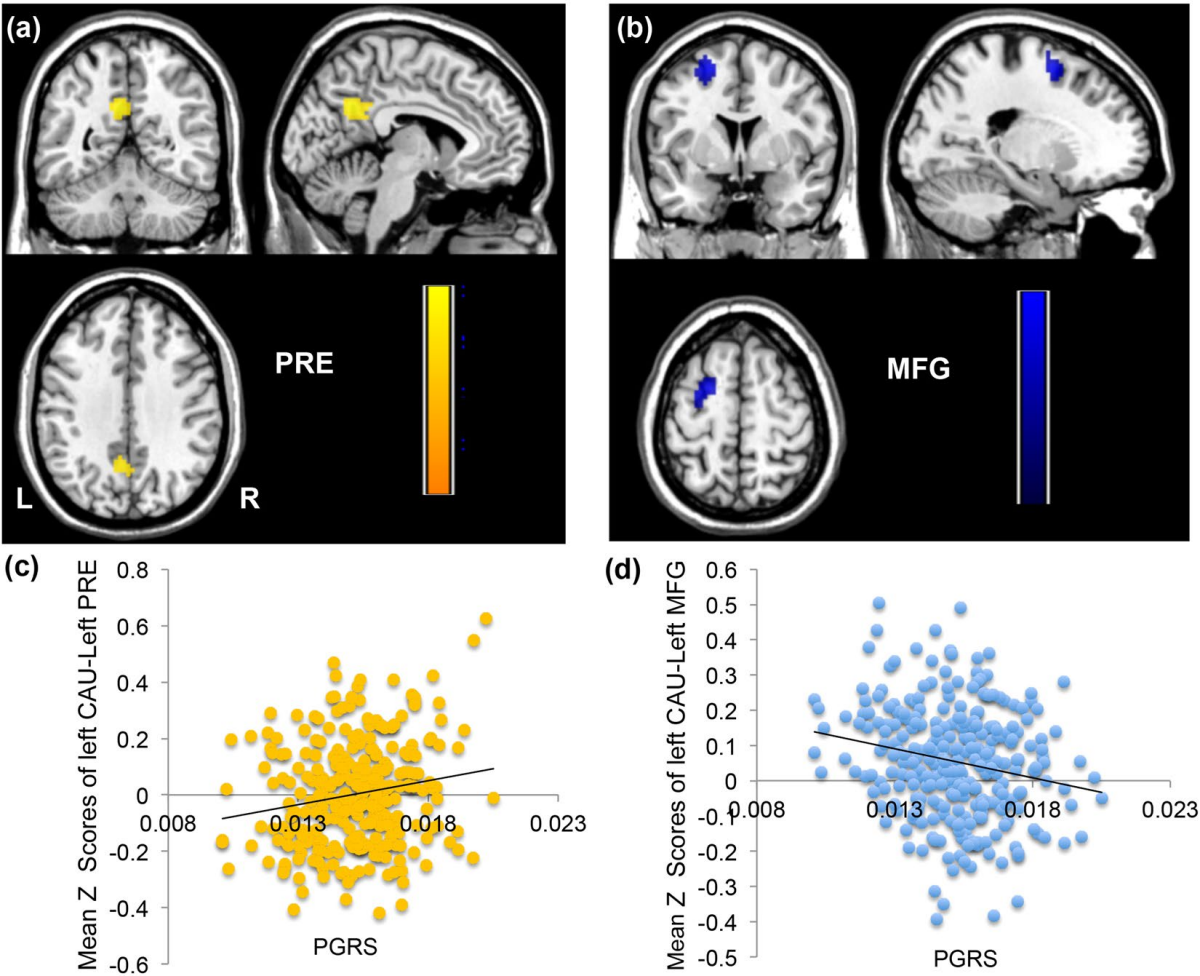
Association of the dopamine gene-related PGRS and functional connectivity with the caudate nucleus. (**a**) and (**c**) show that functional connectivity between the caudate nucleus (CAU) and precuneus (PRE) increases with higher PGRS values (r = 0.188, p = 2 × 10^−3^); (**b**) and (**d**) show that the connection between the caudate and the middle frontal gyrus (MFG) is attenuated as the PGRS values increase (r = −0.205, p = 1 × 10^−3^) (Alphasim corrected p < 0.05, clusters > 79).

**Figure 3:**
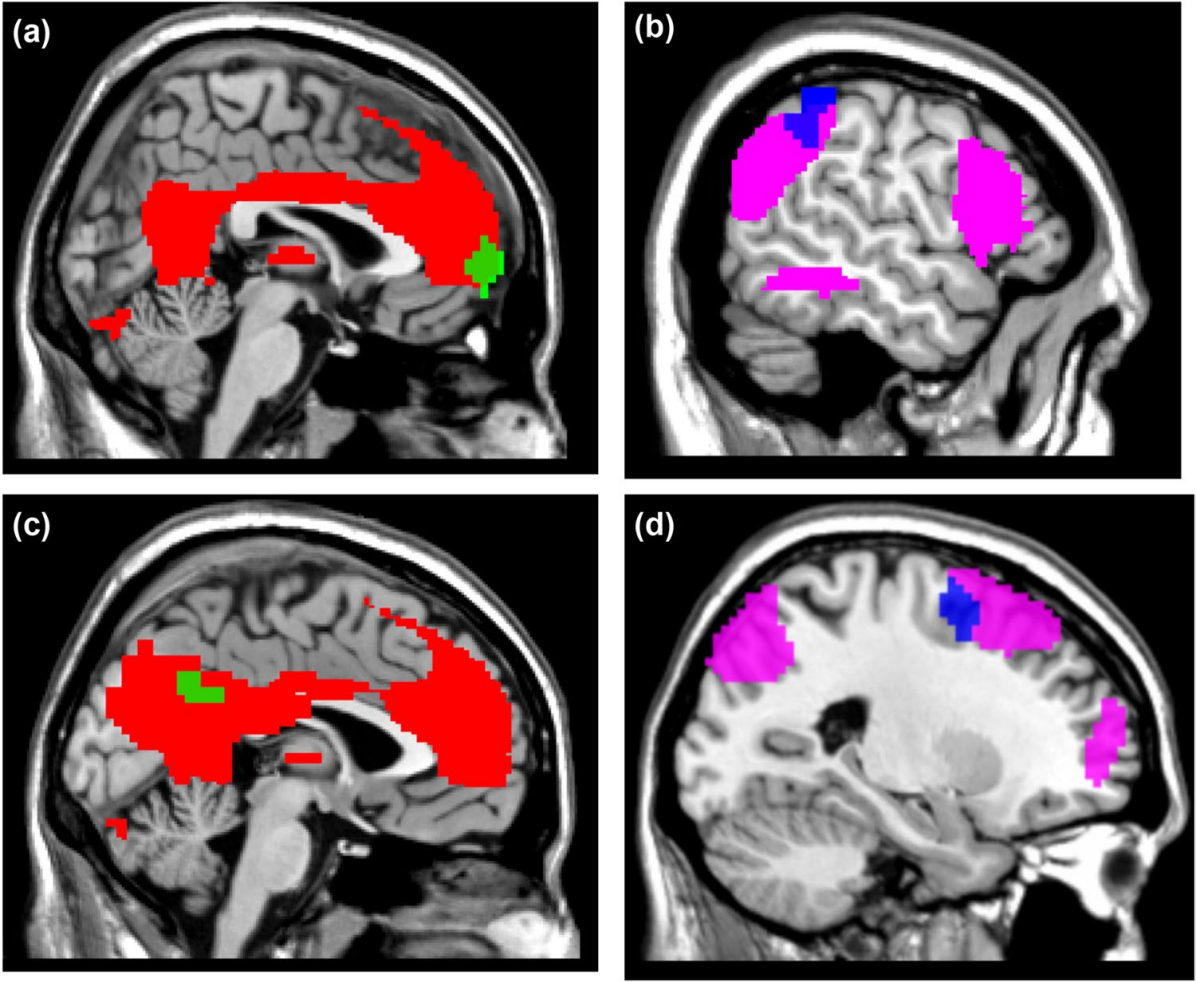
Overlaps of the significant clusters of the MPFG (**a, green**), the IPL (**b, blue**), the PRE (**c, green**), and the MFG (**d, blue**) with their corresponding functional network masks. The red mask represents the default mode network (**a**,**c**); the violet mask represents the fronto-parietal network (**b**,**d**).

In multiple regression analysis, we observed pronounced positive correlations between the PGRS and the functional connectivity between the left putamen and the medial prefrontal cortex (r = 0.214, p = 3.62 × 10^−4^, Fig. 1(a,c)); meanwhile, the functional connectivity with the inferior parietal lobule was anti-correlated with the PGRS (r = −0.219, p = 2.59 × 10^−4^, Fig. 1(b,d));. The PGRS showed a linear positive correlation with the functional connectivity between the left caudate nucleus and the precuneus (r = 0.188, p = 2 × 10^−3^, Fig. 2(a,c)), but it showed an inverse correlation with the functional connectivity between the left caudate and the middle frontal gyrus (r = −0.205, p = 1 × 10^−3^, Fig. 2(b,d)). The overlap between each significant region and its corresponding resting-state functional network demonstrated that regions showing positive correlations were all components of the default mode network (Fig. 3(a,c)), and those that showed anti-correlation were parts of the fronto-parietal network (Fig. 3(b,d)). We did not find any significant correlation between SNP and functional connectivity in the post-hoc tests (all p > 0.05). And we did not observe a polygenic effect of PGRS on the functional connectivity with the ventral striatum in our current sample.

## Discussion

Based on all SNPs in the dopamine genetic pathway that confer risk for schizophrenia, our present study found that dopamine gene-related PGRS showed are markable correlation with the function of the dorsal striatum and with working memory performance. Moreover, the PGRS was positively correlated with the functional connections between brain regions involved in the default mode network and the substructure of the dorsal striatum and was negatively correlated with the functional connections of regions within the fronto-parietal network. These insights elucidate the complex mechanisms by which genomic variation influences schizophrenia-related cognitive performance through its effect on neural circuitry.

We only found that dopamine gene-related PGRS was correlated to functional connectivity with substructures of the dorsal striatum. These results were consistent with previous case-control studies, Dandash *et al*. also found that only the functional connectivity with dorsal striatum was changed in subjects with an at-risk mental state for psychosis, moreover altered functional connectivity was closely related to positive psychotic symptoms^20^. In addition, Wenjin Zhang *et al*.^21^ utilized resting-state fMRI in a group of never-medicated patients with long-term schizophrenia and also found increased spontaneous neural activity in the putamen bilaterally^22^. It is intriguing to speculate that dysfunctions of the dopaminergic system are related to alterations in neural activity, and this might be a relevant pathophysiological mechanism that increases the risk of schizophrenia.

We also observed that the brain areas correlated with the dopamine gene-related PGRS were located in the default mode network and the fronto-parietal network, whereas the putamen and caudate nucleus belong to the salience network^23^. Williamson proposed that anomalous coordination of the default mode network and the task-related network might be a probable etiology of schizophrenia^24^. Subsequently, Menon further introduced the salience network, suggesting that aberrant functional integration and interconnectivity of the default mode network, the central executive network (similar to the task-related network) and the salience network might be the major cause of most psychiatric disorders^25^. The default mode network mainly contains the medial prefrontal cortex extending to the ventral anterior cingulate and the posterior cingulate extending to the precuneus, and the lateral parietal cortex^26^. Dysfunction of the default mode network may lead to cognitive impairments including decreased concentration, deterioration of working memory, and schizophrenia-related positive clinical symptoms such as delusions and hallucinations^27^. Extensive studies have indicated that schizophrenia patients and non-psychiatric relatives exhibited hyper-activation and hyper-connectivity of the default mode network^9,28^. Moreover, weaker task-related suppression of the default mode network is consistently found in both task and resting-state fMRI analysis of schizophrenia patients. Our present results exhibited a consistent trend: for individuals, higher risk of schizophrenia was associated with stronger connections between the dorsal striatum and the default mode network. However, the fronto-parietal network is a task-positive network involved in top-down cognitive control, and its functional connectivity is positively correlated to working memory capacity^29^. Numerous studies have found that brain regions in the fronto-parietal network show decreased task-related activation in multiple-tasks^30,31^. Resting-state fMRI analyses have also revealed reduced intra- and inter-connectivity of the fronto-parietal network^22,32^. Our findings were consistent with previous reports suggesting that increasing risk of schizophrenia was accompanied by suppressed activity in the task-positive network and increased activity in the task-negative network.

Interestingly, the dorsal striatum showed entirely opposite correlations with the default network and fronto-parietal network. Power and others proposed that the functional organization of the human brain can be mainly divided into processing system, including the visual, sensorimotor and default networks, and control system, including the fronto-parietal, attention and salience networks^33^. The processing system are relatively stable, whereas the control system is involved in completing various dynamic tasks and is therefore more flexible, allowing it to adapt and to manage multiple complex tasks^33,34^. Moreover, in our previous study of resting-state functional connectivity density, the modulation patterns of the dopamine system on these two types of brain systems were opposite^35^. These results are consistent with the regulation model of catecholaminergic signaling on stress-induced brain activity during working memory processing^36^. For the moment, at least, these inverse modulation patterns have been observed in several studies, although we cannot identify the neural mechanism underlying the functional system-dependent modulation of the dopamine system.

Moreover, it is striking that only the left substructure of the dorsal striatum showed a connection with the dopamine gene-related PGRS, possibly due to brain asymmetry. Previous studies have indicated that the levels of dopamine and dopamine transporters and the density of dopamine D2 receptors in presynaptic neurons showed right laterality in healthy controls, whereas these physiologic asymmetries are reduced in schizophrenia patients^37^. A study by Muller and others showed that schizophrenia patients exhibited weaker left-against-right asymmetry in the caudate nucleus than did healthy controls^38^. This might indicate that the right laterality of striatal dopamine metabolism is always connected with the left-against-right asymmetry of striatal hemispheric specialization in healthy subjects and that this laterality is diminished in schizophrenia^38^. Thus, the structure and function of the left dorsal striatum are sensitive to risk for schizophrenia.

Several deficiencies in this study cannot be ignored, particularly the following: firstly, the uncertainty of imputation for un-genotyped SNPs may be due to different choices regarding parameters such as the slide window, sample size and imputation methods; secondly, the polygenic risk score is calculated based on the assumption of additive gene effects without considering gene interaction; lastly, although the largest GWAS of schizophrenia to date was used, most of these subjects are of European ancestry. Previous study have found that the association between several SNPs in ZNF804A, NRGN and schizophrenia were different across distinct ethic population, the significance level in Asian populations were not across the whole genome^19,39,40^. However, although several genetic variants showed genetic heterogeneity among distinct populations, the combination of these variants did not statistically differ between Asian and European groups^41^. And we initially defined a loose threshold to include as many significant SNPs conferring risk for schizophrenia as possible, so our research should remain meaningful.

In conclusion, the dopamine gene-related polygenic risk for schizophrenia score has predictive utility for the dorsal striatal volume, functional connectivity within the dopaminergic circuits and working memory performance. Furthermore, we only included genes associated with the dopaminergic genetic pathway in order to minimize the confounding effects of other types of neurotransmitters on schizophrenia-related brain regions. This approach may help us to clarify the underlying neurobiological mechanism of “the dopamine hypothesis” for schizophrenia, and provide some evidences regarding the polygenic risk for schizophrenia. Future studies are warranted to investigate the effects of different subgroups of dopamine functioning, which might help us elucidate the effect of dopaminergic activity in the risk for schizophrenia.

## Materials and Methods

### Subjects

Three hundred twenty-three healthy young Chinese subjects (mean age: 22.7 ± 2.5 years; age range = 18–31 years; 157 males) were recruited. The exclusion criteria were identical to our previous study that used the same database^42^. We carefully asked subjects to ensure that had no personal or family history of neurological or psychiatric disease, head injury, psychiatric treatment, drug or alcohol abuse, hypothyroidism or other mental diseases and no contraindications to magnetic resonance imaging (MRI) screening. All of the participants and their first-degree relatives had no history of psychiatric disease. The study was approved by the local Medical Research Ethics Committee of Tianjin Medical University. All experimental methods were performed in accordance with the relevant guidelines and regulations, and written informed consents were obtained from each subject. N-back working memory tasks^43^ were used to assess working memory before MRI acquisition. The letter 2-back and 3-back tasks were described previously^44^. Briefly speaking, a series of letters were sequentially presented on the screen, each subject was asked to view and judge whether the letter of the current screen was identical to the one displayed two trials (2-back) and three trials (3-back) back. The N-back task contained three blocks (30 trials each block). Before the formal tasks, participants were instructed and took a practice. The numbers of correct and false responses were recorded to compute the accuracy as the index of working memory. Eight subjects were discarded from further analysis due to missing genotype data (see Table 1 for demographic characteristics).

**Table 1:** Demographic Characteristics of the participants.

	Demographic Characteristics
Number of subjects	315
Male (%)	48.9%
Age (Mean $\pm \,{\rm{SD}}$)	22.73 ± 2.50
Age range	18–31 years

### DNA extraction and genotyping

DNA was extracted from venous whole blood samples treated with ethylene diamine tetraaceticacid (EDTA) as an anticoagulant by using the EZgene Blood gDNAMiniprep Kit (BioMiga, San Diego, CA). Genotyping used the standard Illumina genotyping protocol (Illumina) on Illumina Human OmniZhongHua-8 Bead Chips. The following quality control procedures were carried out using PLINK version 1.07^45^. The SNPs were removed where the call rate was <95%, if the minor allele frequency was <1%, or if the χ^2^ test of Hardy-Weinberg equilibrium was <10^−3^. In addition, individuals were removed from the study if their missing genotype rates were >5%, if the estimate of pairwise identity-by-descent (IBD) was >0.1875, or if the gender identified by X chromosome markers differed from the patient records. To control for population stratification, we ascertained population structure using principal component analyses (PCA) in EIGENSTRAT software (http://www.hsph.harvard.edu/alkes-price/files/eigensoftfaq.htm)^46,47^ on autosomal SNPs with the HapMap phase 3 reference data set^48^. Prior to the PCA, we removed chromosomal regions of long-range linkage disequilibrium (LD) and retained SNPs that were in low LD with each other. After extracting 10 principal components, we removed the samples outside 6 standard deviations. The data were then imputed using SHAPEITv2 (https://mathgen.stats.ox.ac.uk/genetics_software/shapeit/shapeit.html)^49^ and IMPUTE2 (https://mathgen.stats.ox.ac.uk/impute/impute_v2.html)^50^ with the 1000 Genomes Phase 1 reference dataset. Further analyses focused on 315 subjects with more than 7 million autosomal SNPs, whose imputation quality scores were greater than 0.8.

### Computation of dopamine genes-related PGRS

We used the “score” function implemented in PLINK to compute the dopamine genes-related polygenic schizophrenia-risk score. The PGRS was calculated according to the method developed by Purcell *et al*.^51^. Our analyses focused on those genes involved in dopamine functioning: (i) synthesis (tyrosine hydroxylase (TH), dopa decarboxylase (DDC), and vesicular monoamine transporter (VMAT)); (ii) uptake and metabolism (dopamine transporter (DAT) and catechol-O-methyl transferase (COMT)); (iii) receptors (D1 and D5 receptors in D1 family, as well as D2, D3 and D4 in D2 family)^52^. We evaluated all available SNPs both occurring in the area 5000 bp upstream and downstream of each candidate gene and meeting a significance level of p ≤ 0.05 in the multi-stage schizophrenia GWAS study^19^ because this was the level that most efficiently discriminated individuals with and without schizophrenia. The SNPs were pruned using the “clump” utility in PLINK with a cutoff of r^2^ = 0.50 within a 250 kb window to ensure that the polygenic effects come from independent SNPs in linkage disequilibrium with each other (Supplementary table 1 showed the SNPs and weights used to generate the PGRS).

### Image acquisition and processing

Images were collected using a single 3T GE scanner (SIGNAHDX3.0 T scanner; GE Healthcare; Milwaukee, WI, USA). A single-shot, T2*-weighted gradient echo, echo-planar-imaging sequence (TR = 2000 ms, TE = 30 ms, no gap, voxel size = 3.75 mm × 3.75 mm × 4.0 mm, FOV = 240 × 240 mm^2^, matrix = 64 × 64, flip angle = 90°, 40 slices, 180 volumes) was used to obtain resting-state functional magnetic resonance imaging data (fMRI). All subjects were requested to relax and to not fall asleep.

Two experienced radiologists inspected all the raw MRI data. Preprocessing of functional images was performed using DPARSFA (Data Processing Assistant for Resting-State fMRI Advanced Edition, http://www.restfmri.net/forum/DPARSF) in statistical parametric mapping software (SPM8, http://www.fil.ion.ucl.ac.uk/spm). The preprocessing included discarding the first 10 volumes, slice timing, head motion correction, spatially normalizing to the Montreal Neurological Institute (MNI) template, resampling to 3 × 3 × 3 mm^3^, smoothing with a 6 mm Gaussian kernel, temporal band-pass filtering, and linear regression to remove the influence of nuisance signals including head motion parameters and white matter, cerebrospinal fluid and global signals. Then, we discarded 14 participants whose maximum displacement in any of the cardinal directions (x, y, z) was greater than 2 mm or whose maximum spin (x, y, z) was greater than 2°. In the end, 286 subjects remained in the functional connectivity analysis.

The seed regions (bilateral caudate nucleus, putamen and ventral striatum) were extracted based on the Oxford-GSK-Imanova Structural-anatomical Striatal Atlas^53^, and the masks for the seed regions were resampled to 3 × 3 × 3 mm^3^ in MNI space. We calculated the Pearson’s correlation coefficient between the average time series of the seed regions and those of all the voxels of the whole brain. Then, we transformed the r correlation into a Z score via Fisher’s r-to-z transformation in order to obtain Z maps for each subject. Next, one-sample *t*-tests were used to determine the main functional connectivities in for each striatal sub-region. Then, we used the masks of the one-sample *t*-tests results for subsequent multiple regression analysis.

### Statistical analysis

Voxel-wise multiple regression analysis was implemented in SPM8 to investigate the correlation between dopamine gene-related PGRS and the seed-based functional connectivity. To correct for multiple statistical testing, we maintained a cluster-level false-positive detection rate at p < 0.05 using a voxel-level threshold of p < 0.001 (Supplementary Figures S1 and S2) and a voxel-level threshold of p < 0.005 with a cluster extent determined by Monte Carlo simulations (n = 5000 iterations). We accomplished this step using Alphasim implemented in the Data Processing & Analysis for Brain Imaging V2.3 (DPABI, http://rfmri.org/dpabi)^54^. To determine the functional networks to which brain regions with significant correlation with the PGRS belonged, we performed group independent component analysis (group ICA) to separate the blood oxygen level-dependent signal into independent components using theprogram Group ICA of fMRI Toolbox (GIFT) (http://icatb.sourceforge.net/). Based on a previous study by Simth *et al*.^55^, we identified nine resting-state networks, including the default mode network and the fronto-parietal network, and we obtained the mean component masks.

In addition, partial correlation analysis was performed to test the correlation between the PGRS and working memory performance as well as functional connectivity with striatal sub-regions with age, gender and the first three genetic principal components as covariates. We further did post-hoc tests to determine which SNPs might be the most responsible for the observed effect using PLINK version 1.07.

### Data availability

The datasets analyzed during the current study are available from the corresponding author on reasonable request.

## Electronic supplementary material

Supplementary materials
