# Supplementary material for: Multilocus genetic profile in dopaminergic pathway modulates the striatum and working memory
Source: Sci Rep. 2018 Mar 29;8:5372. doi: 10.1038/s41598-018-23191-y (PMC5876382; doi:10.1038/s41598-018-23191-y)

**Multilocus genetic profile in dopaminergic pathway modulates the striatum and working memory**

Chao Wang^1,2,+^, Bing Liu^3,4,+^, Xiaolong Zhang^3,4^, Yue Cui^3,4^, Chunshui Yu^6,*^, Tianzi Jiang^2,3,4,5,7,*^

^1^ Shenzhen Key Laboratory of Affective and Social Cognitive Science, College of Psychology and Sociology, Shenzhen University, Shenzhen, 518060, China

^2^ Key Laboratory for NeuroInformation of Ministry of Education, School of Life Science and Technology, University of Electronic Science and Technology of China, Chengdu 610054, P. R. China

^3^ Brainnetome Center, Institute of Automation, Chinese Academy of Sciences, Beijing, China

^4^ National Laboratory of Pattern Recognition, Institute of Automation, Chinese Academy of Sciences, Beijing, China

^5^ CAS Center for Excellence in Brain Science, Institute of Automation, Chinese Academy of Sciences, Beijing, China

^6^ Department of Radiology, Tianjin Medical University General Hospital, Tianjin, China

^7^ The Queensland Brain Institute, University of Queensland, Brisbane, Australia

^+^ Chao Wang and Bing Liu contributed equally to this work and should be considered co-first authors.

^*^**Address correspondence to**:

Tianzi Jiang, Brainnetome Center, Institute of Automation, Chinese Academy of Sciences, Beijing 100190, China

Phone: +86-10-8254 4778; Fax: +86-10-8254 4777

Email: [jiangtz@nlpr.ia.ac.cn](mailto:jiangtz@nlpr.ia.ac.cn)

Or

Chunshui Yu, Department of Radiology, Tianjin Medical University General Hospital, No. 154, Anshan Road, Heping District, Tianjin 300052, China.

Fax: +86 22 6036 2990

E-mail: [chunshuiyu@vip.163.com](mailto:chunshuiyu@vip.163.com)

**Supplementary Table 1** SNPs and risk alleles used to calculate a genetic risk score.

| Gene | SNP | Risk allele | OR |
| --- | --- | --- | --- |
| DRD3 | rs62268961 | G | 1.031481 |
| DRD5 | rs2030287 | T | 1.04582 |
| DRD5 | rs2076907 | G | 1.062157 |
| DRD1 | rs11747886 | A | 1.03241 |
| DDC | rs11771818 | G | 1.035829 |
| DDC | rs732215 | C | 1.033549 |
| DDC | rs11575375 | G | 1.022756 |
| DDC | rs1470750 | G | 1.036764 |
| DDC | rs3779074 | G | 1.027982 |
| DBH | rs3025373 | G | 1.039879 |
| VMAT2 | rs11197931 | C | 1.042688 |
| VMAT2 | rs363226 | G | 1.028605 |
| VMAT2 | rs363285 | C | 1.026546 |
| DRD4 | rs28575104 | G | 1.024191 |
| DRD2 | rs2440390 | T | 1.03749 |
| DRD2 | rs78022226 | G | 1.075951 |
| DRD2 | rs76208665 | G | 1.040702 |
| DRD2 | rs12574471 | T | 1.04467 |
| DRD2 | rs17529477 | G | 1.057496 |
| DRD2 | rs4245147 | T | 1.03904 |
| DRD2 | rs77195172 | T | 1.06801 |
| DRD2 | rs55697087 | G | 1.039566 |
| DRD2 | rs80014933 | C | 1.073364 |
| DRD2 | rs7131056 | A | 1.04091 |
| DRD2 | rs4630328 | G | 1.066416 |
| COMT | rs143721127 | G | 1.135306 |
| COMT | rs174693 | A | 1.03303 |
| COMT | rs73880029 | C | 1.060355 |
| COMT | rs4646312 | C | 1.021941 |
| COMT | rs174696 | T | 1.02881 |
| COMT | rs887199 | G | 1.031481 |

OR=odds ratio.

**Supplementary Figure S1** **Association of the dopamine gene-related PGRS and functional connectivity with the putamen.** (a) and (b) show that the functional connectivity between putamen (PUT) and medial prefrontal cortex (MPFG) increases with higher PGRS values (r = 0.219, p = 2.12×10^-4^) (uncorrected voxel-level p < 0.001, Alphasim corrected *p* < 0.05, clusters > 19).


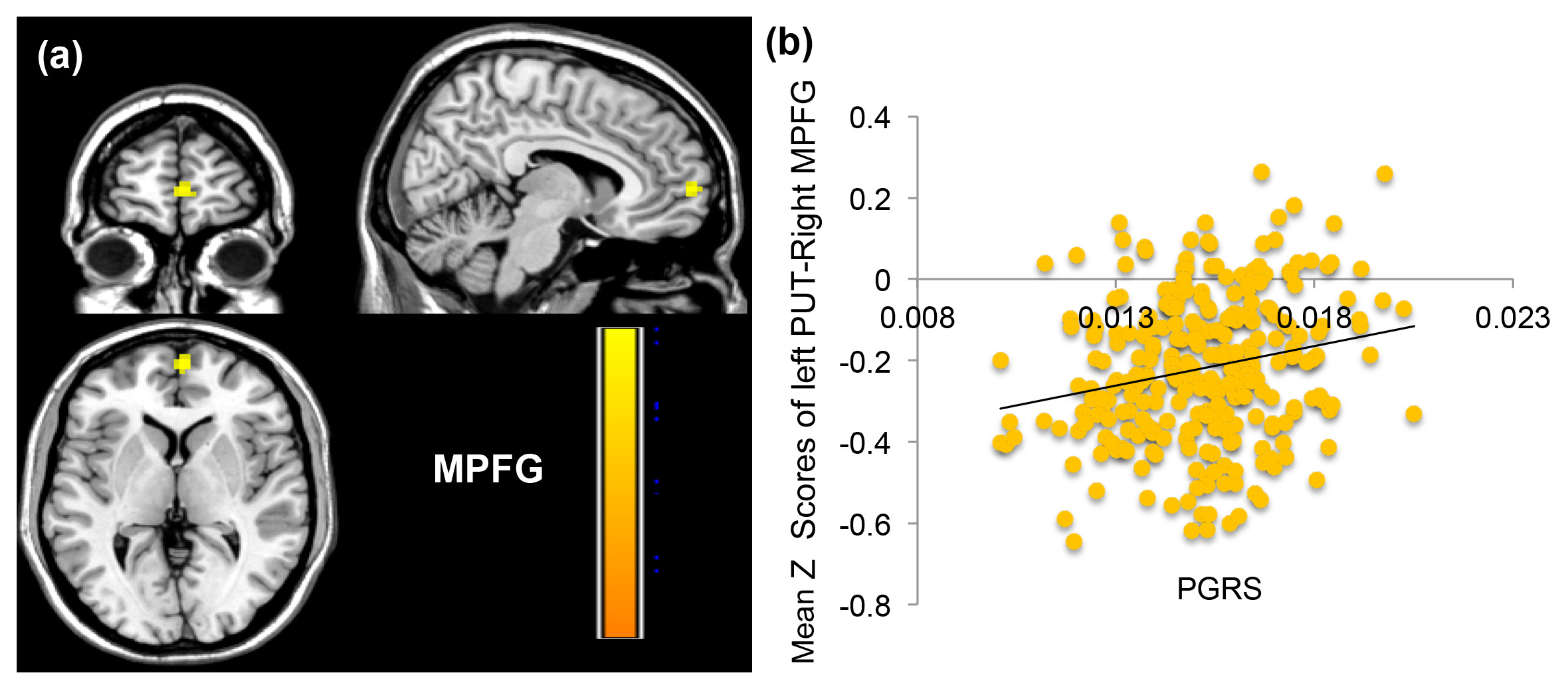


**Supplementary Figure S2 Association of the dopamine gene-related PGRS and functional connectivity with the caudate nucleus.** (a) and (b) show that functional connectivity between the caudate（CAU） and the middle frontal gyrus (MFG) is attenuated as the PGRS values increase (r = -0.215, p = 2.79×10^-4^) (uncorrected voxel-level p < 0.001, Alphasim corrected *p* < 0.05, clusters > 19).


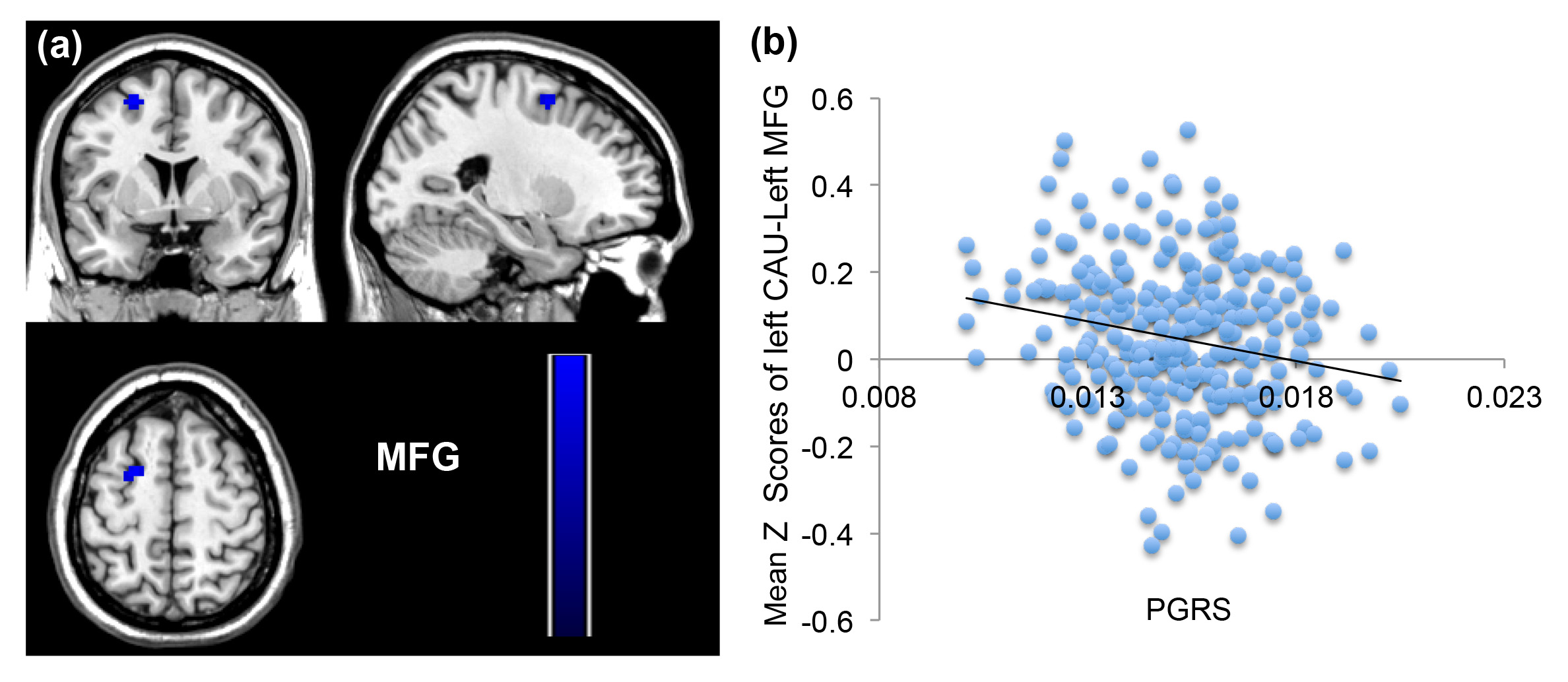

Supplement: Supplementary file 1 — Supplementary materials [file 41598_2018_23191_MOESM1_ESM.doc]
